# The Possibility of DNA Methylation for Precision Medicine in Colorectal Cancer

**DOI:** 10.14789/ejmj.JMJ25-0002-R

**Published:** 2025-05-09

**Authors:** KIICHI SUGIMOTO

**Affiliations:** 1 Department of Coloproctological Surgery, Juntendo University Faculty of Medicine, Tokyo, Japan; 1 Department of Coloproctological Surgery, Juntendo University Faculty of Medicine, Tokyo, Japan

**Keywords:** colorectal cancer, precision medicine, DNA methylation, liquid biopsy

## Abstract

I (the author) received the 44th Juntendo Medical School Alumni Association Academic Encouragement Award in May 2022. After graduating from Juntendo University School of Medicine in 2002, I trained in the Department of Coloproctological Surgery at Juntendo University, led by Professor Toshiki Kamano and Professor Kazuhiro Sakamoto. I then studied DNA methylation in cancer at Johns Hopkins University in Maryland in the United States of America from December 2013 to July 2016. I worked in the laboratory of Professor Malcolm V Brock, a thoracic surgeon, in the Department of Thoracic Surgery. The subject of this translational research was ‘Epigenetics’, and specifically DNA methylation in lung and esophageal cancer. Epigenetics is the study of potentially heritable changes in gene expression that do not involve changes to the underlying DNA sequence, while genetics involves changes to the underlying DNA sequence. In epigenetic alterations, methyl marks added to certain bases repress gene activity by tightly packing the chromatin. Recently, there have been many attempts to apply epigenetics to clinical diagnosis, treatment and prevention.

Colorectal cancer is a global cause of death and an increasingly common disease worldwide. To predict and improve long-term outcomes in CRC, a wide variety of perioperative biomarkers, including imaging markers, blood markers, and pathological and molecular markers, have been reported as prognostic factors. This review includes a description of three of our previous studies on DNA methylation and summarizes the potential clinical usefulness of the findings in precision medicine for colorectal cancer.

## Introduction

I (the author) received the 44th Juntendo Medical School Alumni Association Academic Encouragement Award in May 2022. I would like to thank everyone who granted me this award. After graduating from Juntendo University School of Medicine in 2002, I trained in the Department of Coloproctological Surgery at Juntendo University, led by Professor Toshiki Kamano and Professor Kazuhiro Sakamoto. In 2011, I obtained a Ph.D. degree in clinical research under the supervision of Professor Sakamoto. I then studied DNA methylation in cancer at Johns Hopkins University in Maryland in the United States of America from December 2013 to July 2016. I worked in the laboratory of Professor Malcolm V Brock, a thoracic surgeon, in the Department of Thoracic Surgery. The subject of this translational research was ‘Epigenetics’, and specifically DNA methylation in lung and esophageal cancer.

## Background

Colorectal cancer (CRC) is a global cause of death and an increasingly common disease worldwide^1, 2)^. To predict and improve long-term outcomes in CRC, a wide variety of perioperative biomarkers, including imaging markers, blood markers, and pathological and molecular markers, have been reported as prognostic factors^3)^.

Epigenetics is the study of potentially heritable changes in gene expression that do not involve changes to the underlying DNA sequence, while genetics involves changes to the underlying DNA sequence^4)^. Genetic and epigenetic abnormalities are linked with each other, which can lead to cancer development and progression^5)^. In epigenetic alterations, methyl marks added to certain bases repress gene activity by tightly packing the chromatin^6)^, and there have been many recent attempts to apply epigenetics to clinical diagnosis, treatment and prevention^7, 8)^. This review includes a description of three of our previous studies on DNA methylation and summarizes the potential clinical usefulness of the findings in precision medicine for CRC.

### Research #1: CHFR promoter methylation is predictive of response to irinotecan-based systemic chemotherapy in colorectal cancer

Checkpoint with fork head and ring finger domains (CHFR) promoter methylation has been linked to favorable outcomes of irinotecan-based chemotherapy for advanced or metastatic CRC^9)^. However, there has been little exploration of the clinical implications of CHFR methylation status in such cases. In this work, CHFR promoter methylation was shown to be correlated with the efficacy of irinotecan-based chemotherapy for advanced or metastatic CRC^10)^.

The study used a training cohort of 44 frozen colorectal tumor tissues from CRC cases treated with surgery at Juntendo University Hospital between 2017 and 2020, and a test cohort of frozen colorectal tumor tissues from 49 CRC cases treated with at least six cycles of irinotecan-based systemic chemotherapy for advanced or metastatic CRC at Juntendo University Hospital between 2011 and 2019. CHFR promoter methylation data were collected from the training and test sets.

**Figure 1:**
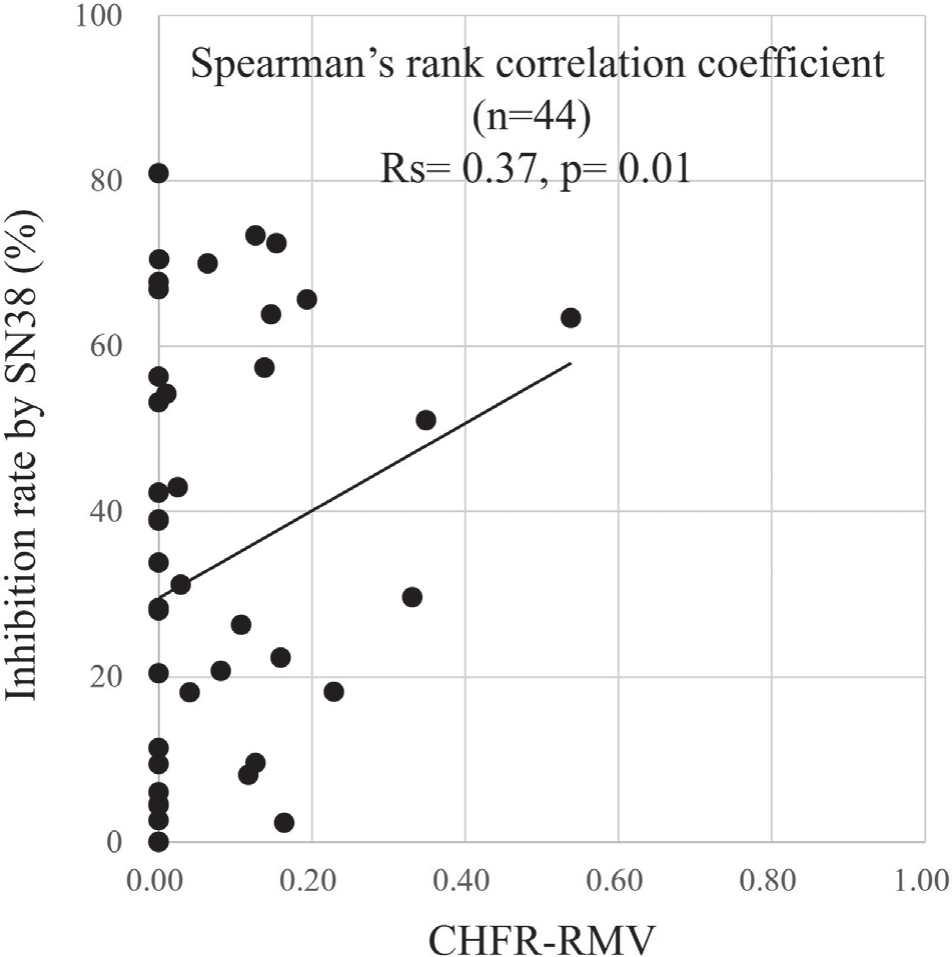
Correlation of CHFR-RMV and inhibition rate by SN38 In the histoculture drug response assay (HDRA), CHFR-relative methylation value (RMV) in the training cohort was significantly correlated with inhibition by SN38 (Rs = 0.37, p = 0.01).

A histoculture drug response assay (HDRA)^11)^ and quantitative methylation-specific PCR (qMSP)^12, 13)^ were performed. For quantification of methylation as the relative methylation value (RMV), each methylation detection replicate was normalized to the mean value for B-ACTIN (ACTB)^12)^. In the HDRA, CHFR-RMV in the training cohort was significantly correlated with inhibition by SN38 (Rs = 0.37, p = 0.01) (Figure 1), but not with inhibition by 5-FU (Rs = 0.21, p = 0.17). The median SN38 inhibition rate was 30.4% (0.0%-80.9%). The chemosensitivity of tumors in the HDRA was defined as positive based on ≥ 50% inhibition^14, 15)^, and a cut-off of 0.0001 for CHFR-RMV at ≥ 50% inhibition gave the largest AUC (AUC = 0.703, p = 0.01). The training cohort tested was then divided into CHFR-RMV high (n = 26) and low (n = 18) cases, based on this cut-off. In the HDRA, the median inhibition rate using SN38 was significantly higher in CHFR-RMV high cases [47.0% (2.3%-80.9%) vs. 10.4% (0.0%-66.9%), p = 0.002]. Univariate analysis of clinicopathological factors indicated that the location of the primary tumor differed significantly between the groups (p = 0.0001), with CHFR-RMV high cases more likely to have right-sided CRC.

**Figure 2:**
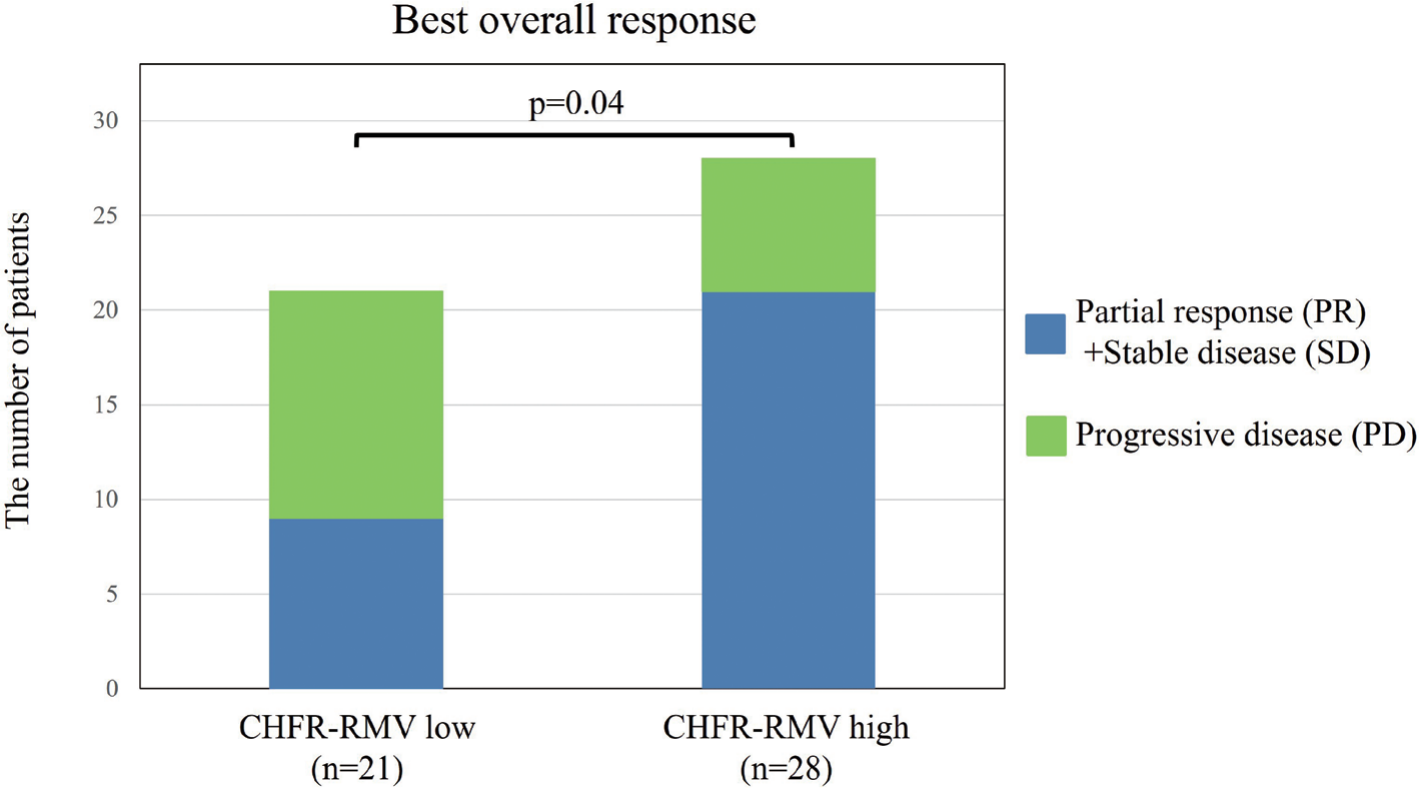
Overall responses to irinotecan-based systemic chemotherapy in CHFR-RMV high and low cases CHFR-RMV high cases had a trend for a better overall response (p = 0.07) and significantly better disease control (PR + SD) (n = 21, 75.0% vs. n = 9, 42.9%, p = 0.04).

**Figure 3:**
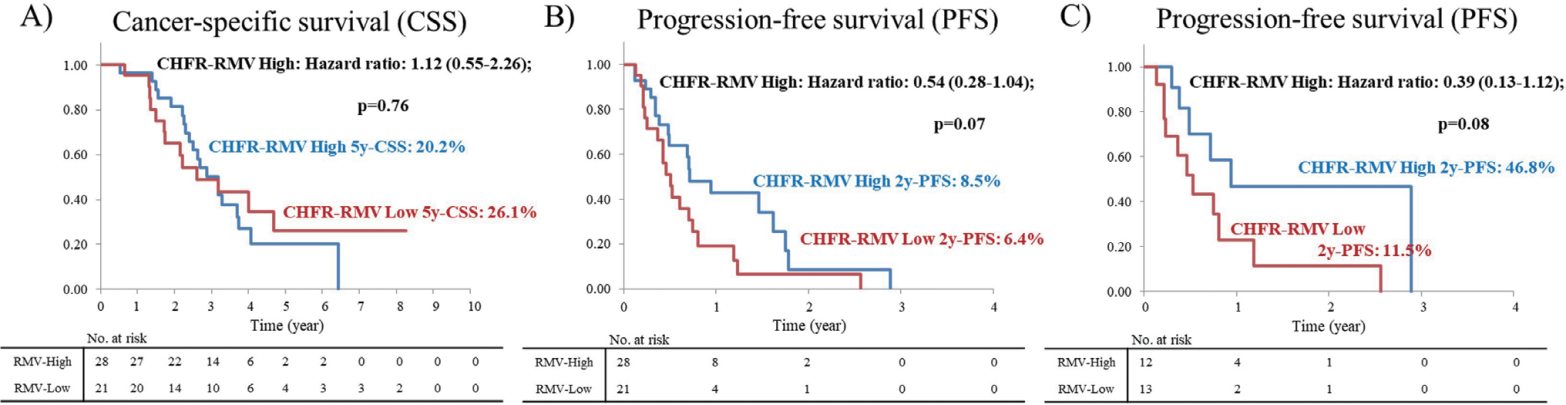
Long-term outcomes in the test cohort Long-term outcomes in the test cohort showed no significant difference in cancer-specific survival (CSS) from initial diagnosis of advanced or metastatic CRC in CHFR-RMV high and low cases [CHFR-RMV high: HR = 1.12 (0.55-2.26), p = 0.76] (Figure 3A). However, CHFR-RMV high cases showed a trend for improved progression-free survival (PFS) from initiation of systemic chemotherapy with irinotecan [HR = 0.54 (0.28-1.04), p = 0.07] (Figure 3B). This trend persisted in patients treated with first-line systemic chemotherapy with irinotecan [HR = 0.39 (0.13-1.12), p = 0.08] (Figure 3C).

Overall responses to irinotecan-based systemic chemotherapy in CHFR-RMV high and low cases were then examined in the test cohort. The following rates (CHFR-RMV high vs. low) were found for partial response (PR) (n = 8, 28.6% vs. n = 3, 14.3%), stable disease (SD) (n = 13, 46.4% vs. n = 6, 28.6%) and progressive disease (PD) (n = 7, 25.0% vs. n = 12, 57.1%). CHFR-RMV high cases had a trend for a better overall response (p = 0.07) and significantly better disease control (PR + SD) (n = 21, 75.0% vs. n = 9, 42.9%, p = 0.04) (Figure 2). A similar analysis in cases treated with first-line systemic chemotherapy with irinotecan also showed a trend for better disease control in the CHFR-RMV high group (83.3% vs. 46.2%, p = 0.10). Long-term outcomes in the test cohort showed no significant difference in cancer-specific survival (CSS) from initial diagnosis of advanced or metastatic CRC in CHFR-RMV high and low cases [CHFR-RMV high: hazard ratio (HR) = 1.12 (0.55-2.26), p = 0.76] (Figure 3A). However, CHFR-RMV high cases showed a trend for improved progression-free survival (PFS) from initiation of systemic chemotherapy with irinotecan [HR = 0.54 (0.28-1.04), p = 0.07] (Figure 3B). This trend persisted in patients treated with first-line systemic chemotherapy with irinotecan [HR = 0.39 (0.13-1.12), p = 0.08] (Figure 3C).

The significant correlation of CHFR promoter methylation with the clinical response of irinotecan- based systemic chemotherapy included clinical benefits for long-term outcomes (i.e., PFS). The association of CHFR promoter methylation with the response to irinotecan may be due to the role of CHFR in protein ubiquitination, as an E3 ubiquitin ligase^16)^. Irinotecan induces DNA damage by inhibition of topoisomerase I^17)^; therefore, repair of this damage is likely to be a mechanism of irinotecan resistance^18)^. A decrease in the CHFR protein level due to DNA methylation impairs ubiquitination of topoisomerase I, and subsequent upregulation of topoisomerase I may increase the sensitivity of cancer cells to irinotecan-induced damage^9)^. Thus, evaluation of CHFR promoter methylation can identify patients who may benefit from irinotecan-based systemic chemotherapy.

### Research #2: Prognostic utility of circulating tumor DNA methylation analysis in Stage IV colorectal cancer

Liquid biopsy involves mainly non-invasive sampling and analysis of non-solid biological tissue (usually blood) for monitoring of diseases such as cancer^19, 20)^. Advances in circulating tumor DNA (ctDNA) detection have enabled use of liquid biopsy assays in clinical practice^20)^. Thus, in this study, the usefulness of locus-specific ctDNA methylation analysis was examined for prediction of long-term outcomes after resection of Stage IV CRC^21)^. The subjects were patients who underwent resection of the primary tumor with and without metastatic organs at Juntendo University Hospital between 2011 and 2020. DNA extracted from plasma samples (i.e., ctDNA) and corresponding frozen colorectal tumors (at the time of resection of the primary tumor) from patients with CRC were subjected to methylation analyses.

Methylation on Beads (MOB) was used for DNA isolation from plasma samples and bisulfite conversion^12, 22, 23)^ for CHFR, SOX11 and CDO1. These genes were chosen for several reasons. CHFR is a mitotic checkpoint and tumor suppressor gene that is inactivated mainly by promoter CpG island methylation, and CHFR methylation is linked to a poor prognosis^10, 16)^. SOX11 is a tumor suppressor gene for which aberrant DNA methylation is found in prostate, gastric and ovarian cancers and chronic lymphocytic leukemia^24)^, with SOX11 methylation associated with poor survival in these cancers^24, 25)^. Abnormal CDO1 regulation through epigenetic changes in the promoter can occur in carcinogenesis, and the extent of this effect is closely related to cancer progression and prognosis^26)^.

**Figure 4:**
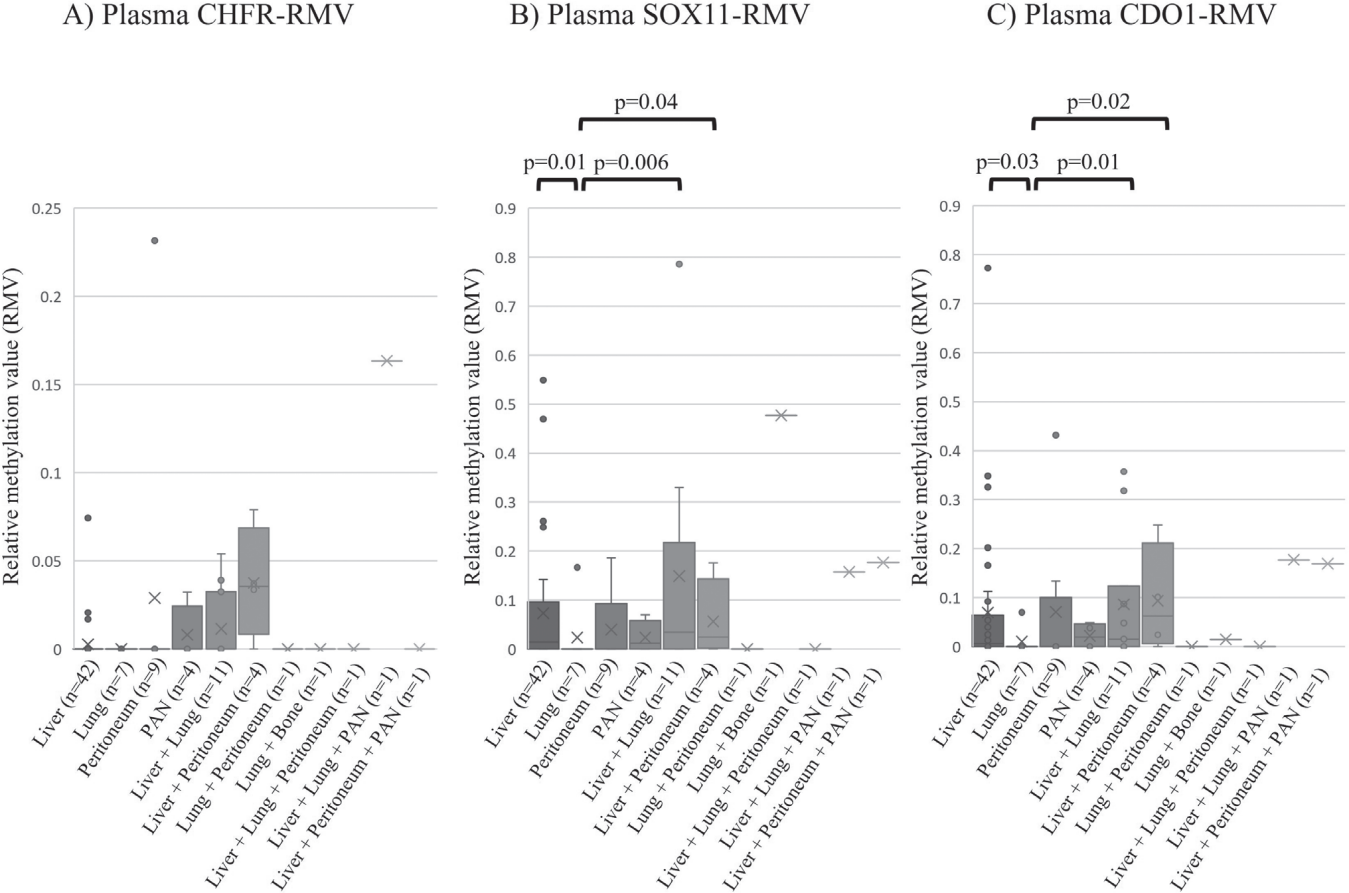
Comparisons of relative methylation values (RMVs) of the three genes at metastatic sites The results showed no significant differences in CHFR-RMV among metastatic sites (Figure 4A); significantly higher SOX11-RMVs in cases with liver metastasis alone (p = 0.01), liver and lung metastasis (p = 0.006), and liver and peritoneum metastasis (p = 0.04) (Figure 4B), and similarly significantly higher CDO1-RMVs at these respective metastatic sites (p = 0.03, p = 0.01, p = 0.02) (Figure 4C), all compared to patients with lung metastasis alone.

The study included 95 patients with adequate DNA quality for methylation analysis. First, RMVs of the three genes at metastatic sites were compared to examine the hypothesis that higher ctDNA methylation may occur in cases with hematogenous metastases (Figure 4). The results showed no significant differences in CHFR-RMV among metastatic sites (Figure 4A); significantly higher SOX11-RMVs in cases with liver metastasis alone (p = 0.01), liver and lung metastasis (p = 0.006), and liver and peritoneum metastasis (p = 0.04) (Figure 4B), and similarly significantly higher CDO1-RMVs at these respective metastatic sites (p = 0.03, p = 0.01, p = 0.02) (Figure 4C), all compared to patients with lung metastasis alone. Correlations of RMVs between plasma samples and cancer tissues were found for CHFR (|r| = 0.458, p < 0.001), but not for SOX11 (|r| = 0.010, p = 0.94) and CDO1 (|r| = 0.011, p = 0.93).

**Figure 5:**
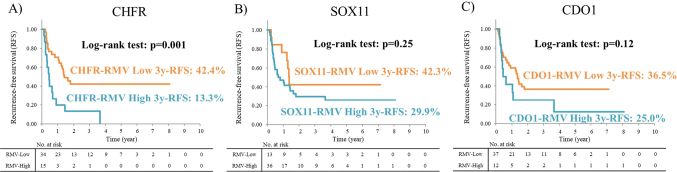
Long-term outcomes for recurrence-free survival (RFS) RFS was significantly poorer in CHFR-RMV high cases than in CHFR-RMV low cases (p = 0.001) (Figure 5A), but did not differ significantly between the high and low SOX11-RMV (p = 0.25) and CDO1-RMV (p = 0.12) cases (Figure 5B, 5C).

Next, long-term outcomes for recurrence-free survival (RFS) were examined. Among patients with resection of the primary tumor with metastatic organs with curative intent, RFS was significantly poorer in CHFR-RMV high cases than in CHFR- RMV low cases (p = 0.001) (Figure 5A), but did not differ significantly between the high and low SOX11- RMV (p = 0.25) and CDO1-RMV (p = 0.12) cases (Figure 5B, 5C). In patients with resection of the primary tumor with metastatic organs with curative intent, in which RFS was significantly worse in CHFR-RMV high cases, the maximum diameter of the primary tumor and the absence of postoperative adjuvant chemotherapy (including any types of regimens such as orally administered 5FU (n = 5) and oxaliplatin-based regimen (n = 32)) were also related to significantly worse RFS in univariate analysis (p = 0.01 and 0.01, respectively). Multivariate analysis using these three variables identified CHFR-RMV [HR = 2.40 (1.18-4.91), p = 0.02] as a significant independent prognostic factor. The maximum diameter of the primary tumor and the absence of postoperative adjuvant chemotherapy were not found to be an independent prognostic factor [HR = 1.91 (0.88-4.14), p = 0.10 and HR = 1.75 (0.79-3.86), p = 0.17, respectively].

A summary of the above analyses suggests worse RFS in CHFR-RMV high cases after resection of the primary tumor with metastatic organs with curative intent, with CHFR-RMV found to be an independent prognostic factor. These findings are consistent with results for the prognostic impact of promoter methylation in CRC cases treated with surgery alone^27)^. Mechanistically, this is based on the relationship of CHFR to cancer progression and metastasis as a tumor suppressor gene that is inactivated by promoter CpG island methylation in solid tumors, since CHFR encodes a checkpoint protein that delays entry into metaphase^9, 28)^. Thus, evaluation of the ctDNA status during treatment or postoperatively may be effective for detection of minimum residual disease (MRD) in the adjuvant setting^29)^. Furthermore, integration of genomic and epigenomic assessment of ctDNA with plasma samples at about 4 weeks post-surgery can improve MRD detection sensitivity^30)^. Thus, use of post-surgery plasma samples for MRD detection is of interest; however, no such samples were available in this study. However, there may be an advantage in evaluation of ctDNA in pre-surgical plasma samples because this allows determination of the indication for resection of the primary tumor with or without metastatic organs in patients with Stage IV CRC. Thus, based on all of the above findings, ctDNA methylation analysis may be useful for predicting the possibility of curative resection and long-term outcomes after resection in Stage IV CRC. CHFR promoter methylation of ctDNA was found to be a negative prognostic factor for RFS in resectable Stage IV CRC in this study (#2) while that of cancer tissue gave a favorable PFS in advanced or metastatic CRC who underwent systemic chemotherapy with irinotecan-based regimen in Research #1. This discrepancy may have resulted from the fact that promoter methylation of CHFR can inhibit its tumor suppressive function as well as it can promote sensitivity of irinotecan. To examine this, the PFS in advanced or metastatic CRC who underwent systemic chemotherapy with other type-based regimen such as oxaliplatin, which is another key drug for CRC, should be investigated. If the PFS was not improved in patients with promoter methylation of CHFR in cancer tissues, this may indicate that promoter methylation of CHFR can be a specific and favorable biomarker to irinotecan while it can basically be a negative prognostic factor for CRC, especially when it appears in ctDNA.

### Research #3: Predictive capability of DNA methylation analysis for histological responses of preoperative treatment for locally advanced rectal cancer

Preoperative chemoradiotherapy (NACRT) for locally advanced rectal cancer (LARC) is performed primarily to suppress postoperative recurrence, but adverse events and long-term outcomes remain uncertain^31)^. In recent years, there has been increased use of total neoadjuvant therapy (TNT), which combines NACRT with preoperative chemotherapy (NAC)^32)^. Despite the increased number of patients with preoperative treatment for LARC to prevent recurrence, predictive modalities for assessing treatment efficacy are lacking and require further study. Therefore, we investigated the predictive utility of DNA methylation analysis as a biomarker for evaluating the effectiveness of preoperative treatment.

The study population comprised patients with preoperative therapy for stage II and stage III LARC (n = 107) who underwent surgery from 2007 to 2020. DNA extraction from formalin-fixed paraffin-embedded specimens and bisulfite conversion were performed^10)^. The Japanese classification of colorectal, appendiceal, and anal carcinoma^33)^ was used to evaluate the histological response to preoperative treatment for LARC. Grade 2 and 3 cases were defined as responders and Grade 0 and 1 cases as non-responders. In the NACRT group, patients received 45-50.4 Gy (1.8 Gy × 25-28 Fr) with oral 5FU. In the NAC group, patients received mFOLFOX therapy (6 cycles) or CAPOX therapy (4 cycles). Surgical resection of LARC was performed one to two months after preoperative treatment.

**Figure 6:**
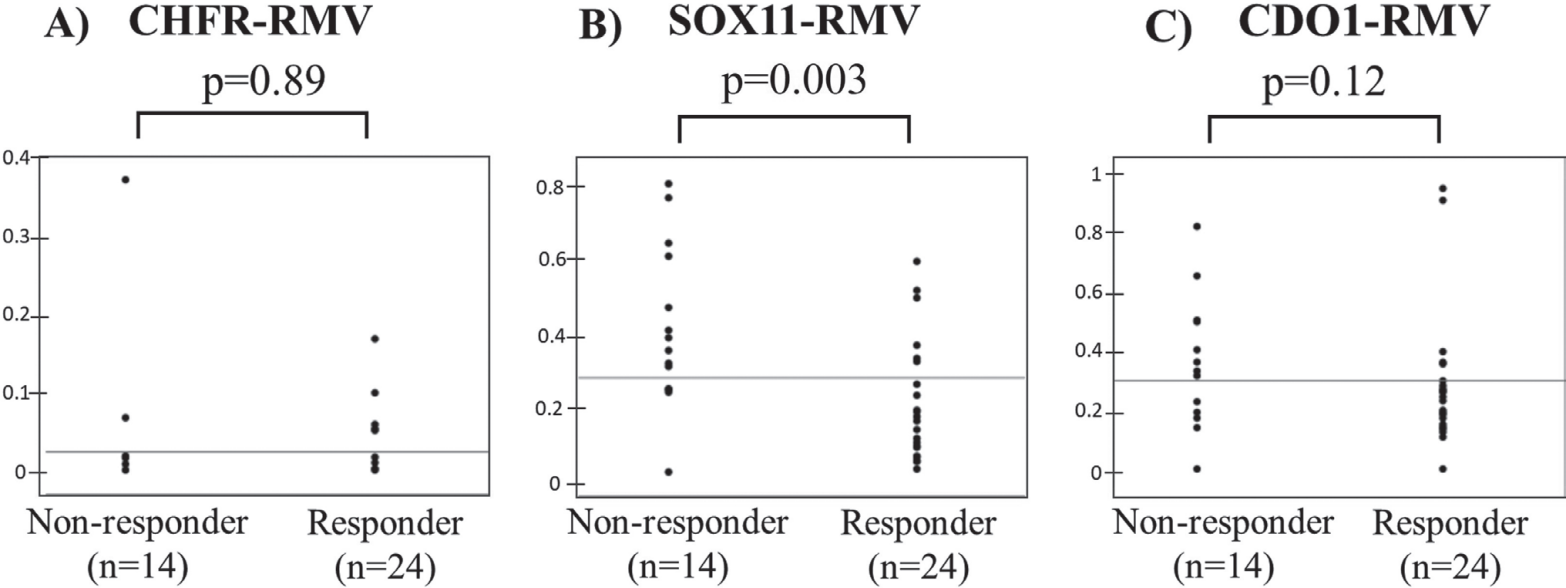
Comparisons of relative methylation values (RMVs) in biopsy specimens before NACRT SOX11-RMV was significantly lower in responders (p = 0.003) (Figure 6A), but that there was no significant difference in CHFR-RMV or CDO1-RMV (p = 0.89, p = 0.12) (Figure 6B, 6C).

**Figure 7:**
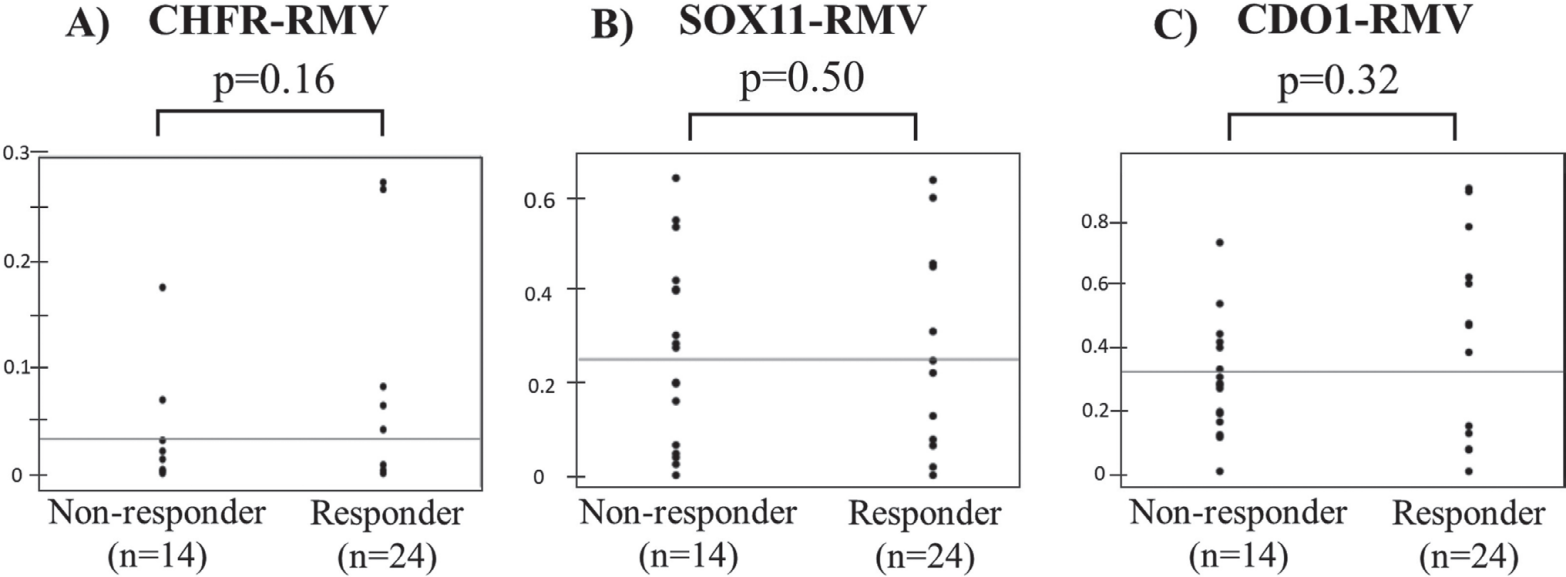
Comparisons of relative methylation values (RMVs) in biopsy specimens before NAC There was no significant difference in RMV for each gene (p = 0.16, p = 0.50, p = 0.32) (Figure 7A, 7B, 7C).

There were 55 patients with NACRT and 52 patients with NAC. The proportion of responders was significantly higher in the NACRT group than in the NAC group (63.6% vs. 36.5%, p = 0.005). A comparison of RMVs in biopsy specimens before NACRT (Figure 6) showed that SOX11-RMV was significantly lower in responders (p = 0.003) (Figure 6A), but that there was no significant difference in CHFR-RMV or CDO1-RMV (p = 0.89, p = 0.12) (Figure 6B, 6C). A similar comparison before NAC (Figure 7) showed no significant difference in RMV for each gene (p = 0.16, p = 0.50, p = 0.32) (Figure 7A, 7B, 7C).

These results show a better histological response after NACRT than after NAC, and suggest that evaluation of SOX11 methylation in biopsied cancer tissue before preoperative treatment may predict the histological response to NACRT. Thus, DNA methylation in cancer tissue may be involved in sensitivity to chemoradiotherapy, which suggests SOX11 methylation analysis may be useful to build preoperative treatment strategies for LARC, such as identification of good candidates for NACRT. SOX11 is a neural transcription factor^34)^ that has been shown to have tumor suppressor functions^35)^. Aberrant DNA methylation of SOX11 is found in most endometrioid endometrial carcinomas and is correlated with clinicopathologic factors in primary endometrial tumors^24)^. The methylation status of SOX11 is also significantly associated with MSI and MLH1 methylation in endometrial tumors^24)^. However, little is known about the function of SOX11 in CRC, and further research is needed. In addition, only a small number of patients were enrolled in our study, and a prospective study in a sufficiently large cohort is needed to investigate the predictive capability of DNA methylation for determining preoperative treatment of LARC.

## Conclusions

The above results show the prognostic and predictive capability of DNA methylation in CRC treatment. A prospective study in a large cohort of CRC cases is needed to validate these findings.

## Funding

The work described in Research #1 and Research #2 was partly supported by Grants-in-aid for Scientific Research from the Japanese Society for the Promotion of Science (17K16580 and 16K19957, respectively).

## Author contributions

Not applicable.

## Conflicts of interest statement

The author declare that there are no conflicts of interest.
